# *In vitro* Inoculation of Fresh or Frozen Rumen Fluid Distinguishes Contrasting Microbial Communities and Fermentation Induced by Increasing Forage to Concentrate Ratio

**DOI:** 10.3389/fnut.2021.772645

**Published:** 2022-01-14

**Authors:** Zhi Yuan Ma, Ju Wang Zhou, Si Yu Yi, Min Wang, Zhi Liang Tan

**Affiliations:** ^1^Chinese Academy of Sciences (CAS) Key Laboratory for Agro-Ecological Processes in Subtropical Region, National Engineering Laboratory for Pollution Control and Waste Utilization in Livestock and Poultry Production, Hunan Provincial Key Laboratory of Animal Nutritional Physiology and Metabolic Processes, Institute of Subtropical Agriculture, The Chinese Academy of Sciences, Changsha, China; ^2^College of Pastoral Agriculture Science and Technology, Lanzhou University, Lanzhou, China

**Keywords:** frozen inoculum, fresh inoculum, *in vitro* technique, rumen fermentation, bacterial community

## Abstract

*In vitro* rumen batch culture is a technology to simulate rumen fermentation by inoculating microorganisms from rumen fluids. Although inocula (INO) are commonly derived from fresh rumen fluids, frozen rumen fluids are also employed for the advantages of storing, transporting, and preserving rumen microorganisms. The effects of frozen INO on microbial fermentation and community may be interfered with by substrate type, which has not been reported. This study was designed to test whether rumen fluid treatments (i.e., fresh and frozen) could interact with incubated substrates. A complete block design with fractional arrangement treatment was used to investigate the effects of INO (fresh or frozen rumen fluids) and concentrate-to-forage ratios (C/F, 1:4 or 1:1) on rumen fermentation and microbial community. The effects of increasing C/F were typical, including increased dry matter (DM) degradation and total volatile fatty acids (VFA) concentration (*P* < 0.001), and decreased acetate to propionate ratio (*P* = 0.01) and bacterial diversity of richness and evenness (*P* ≤ 0.005) with especially higher fermentative bacteria such as genus *Rikenellaceae*_RC, F082, *Prevotella, Bacteroidales*_BS11, *Muribaculaceae*ge, and *Christensenellaceae*_R-7 (*P* ≤ 0.04). Although frozen INO decreased (*P* < 0.001) DM degradation and altered rumen fermentation with lower (*P* ≤ 0.01) acetate to propionate ratio and molar proportion of butyrate than fresh INO, typical effects of C/F were independent of INO, as indicated by insignificant INO × C/F interaction on substrate degradation, VFA profile and bacterial community (*P* ≥ 0.20). In summary, the effect of C/F on fermentation and bacterial diversity is not interfered with by INO type, and frozen INO can be used to distinguish the effect of starch content.

## Introduction

*In vitro* rumen batch culture is a technology to stimulate rumen fermentation. The advantage of this technique is to reduce the cost of animal and related animal welfare issues by testing multiple samples in batch (1). Furthermore, it avoids the effect of host interference factors, such as rumen passage rate and absorption, on the fermentation process (2). Based on the aforementioned advantages, *in vitro* rumen batch culture has been widely employed in ruminant nutrition research to evaluate rumen degradation of feedstuffs (3), the efficiency of rumen-protect protocols (i.e., amino acids and unsaturated fatty acids), and fast screening of methane inhibitors (4–7).

Fresh rumen fluid is commonly employed as INO for *in vitro* fermentation. Increasing evidences show that frozen rumen fluid can be also used as INO. Frozen rumen fluid as INO can easily preserve rumen microorganisms, and allows multiple experiments to be carried out on the same fluids to reduce interexperimental variation (8, 9). Although strong correlations of DM degradation have been observed between fresh and frozen INO (10), evidence also indicates that frozen INO can alter the fermentation pattern with decreased VFA production and shift of fermentation to favor propionate production (11). Such changed rumen fermentation may interact with incubated types of substrates, an effect that has not been investigated.

This study was designed to investigate whether rumen fluid treatment (i.e., fresh or frozen) could interact with incubated substrates that can be helpful to distinguish the effects of contrasting substrates on rumen fermentation. The contrasting substrates used were the increased concentrate-to-forage ratio (C/F), which is characterized by increased DM degradation, shifted fermentation through propionate production, and changed microbial community (2, 12, 13). We hypothesized that both fresh and frozen INO showed a similar pattern concerning the effects of increasing dietary C/F on gas production, fermentation, and bacterial communities. We employed a 2 × 2 factorial design, with an INO of fresh or frozen rumen fluids and substrates with a C/F being 1:4 or 1:1. Interaction between INO and C/F (INO × C/F) was also analyzed.

## Materials and Methods

### Experimental Design

A complete block design with fractional arrangement treatment was used to investigate the effects of INO and C/F on rumen fermentation and bacterial community. Two INO treatments included the fresh or frozen rumen fluids, while two C/F substrates included 1:4 or 1:1. Increased C/F was prepared by replacing rice straw with corn grain meal. We repeated the *in vitro* fermentation for 3 runs, and the run was our replicates.

### Preparation of *In vitro* Inocula

Rumen contents were collected *via* the rumen cannula from two of three fistulated Xiangdong black goats before morning feeding. The goats were fed a mixed diet of rice straw and concentrate (1:1) containing 137 and 380 g/kg DM of crude protein and neutral detergent fiber, respectively. The rumen contents from two goats were filtered through 4-layers cheesecloth individually and then were equally mixed. The mixed rumen fluid was divided into two sterile 50 ml tubes (ϕ = 29 mm, BBI, Shanghai, China). One tube was immediately frozen by liquid nitrogen for about 1 min in liquid nitrogen. The frozen rumen fluid in the liquid frozen tube was removed from liquid nitrogen, and thawed at a 37°C water bath for 10 min to prepare the treatment of frozen INO. The other tube was kept in 37°C water bath to prepare the treatment of fresh INO. Fresh or frozen INO was then mixed with McDougall's buffer (14) at a ratio of 1:4 (vol/vol) to prepare the buffered rumen fluids. All the procedures were conducted under an anaerobic condition with a stream of CO_2_.

### *In vitro* Rumen Batch Incubation and Sample Analysis

About 0.6 g of the substrate was weighed into a 135 ml fermentation bottle, and incubated with 60 ml of buffered rumen fluid under a stream of CO_2_ at 39.5°C. Bottles were immediately placed into the automatic incubation system described by Wang et al. (15), with venting pressure set at 10.0 kPa. The gas production was calculated using the method described by Wang et al. (16).

*In vitro* rumen fermentation was stopped at 48 h. About 2 ml of liquid without visible particles was collected from each bottle and centrifuged at 15,000 g for 10 min at 4°C. The supernatants (1.5 ml) were acidified using 0.15 ml of 25% (w/v) metaphosphoric acid and stored at −20°C for analysis of volatile fatty acids and ammonia according to the method described by Wang et al. (17). About 2 ml of rumen samples were collected after intense hand shaking of the bottle to ensure a representative portion of liquid and particle fractions, immediately put into liquid nitrogen and then stored at −80°C for microbial DNA extraction. The pH was measured immediately by a portable pH meter (Starter 300; Ohaus Instruments Co. Ltd., Shanghai, China) after collecting the samples of VFA and microorganisms. The remaining solid residues were filtered by 37.4 μm aperture gauze and dried at 105°C to constant weight to measure the degradation of the incubated substrates.

Each run was conducted with mixed rumen fluids from two of three donor goats, and repeated three times on different days with different combinations of rumen fluids so that each treatment had three biological replicates. Each run included six culture flasks as technical parallels.

### Bacterial Community Analysis

The microbial DNA was extracted by using a modified RBB+C methodology (18) with sand beating according to Ma et al. (19). The V3–V4 region of 16S rRNA genes of bacteria was amplified by using primers of 341F 5′-CCTAYGGGRBGCASCAG-3′ and 806R 5′-GGACTACNNGGGTATCTAAT-3′ (20). Amplicon sequencing was performed at an Illumina MiSeq PE250 platform by Biozeron, Shanghai, China. The pipeline that generates zero radius OTU (ZOTU) table by using usearch v11 (21) and vsearch 2.17.11 (22) was described in our previous study (23). Taxonomy annotation of representative ZOTUs was conducted by using MOTHUR v1.45.3 (24) with a minimum support threshold of 80% against SILVA NR database v.132 (25). The Shannon index was calculated, and the principal coordinate analysis based on the Bray–Curtis dissimilarity matrix was conducted by using vegan v 2.5 (26).

### Statistical Analysis

All data were analyzed by general linear model using the *lm()* procedure of *R* v 4.0 (27), which was expressed as follow:


$$\begin{array}{l} Y_{ijk}=\mu +INO_{i}+C/F_{j}+INO_{i}\;\times \;C/F_{j}+RUN_{k}+\;e_{ijk} \end{array}$$

where *Y*_*ijk*_ is the response, μ is the general mean, INO is the fixed effect of inocula (*i* = 2), C/F is the fixed effect of C/F (*j* = 2), RUN is the fixed effect of run (*k* = 3), and the *e*_*ijk*_ is the random error term.

Relative abundances of bacteria were commonly deemed non-normal, and then arcsine transformed before fitting the linear model. A non-parametric permutational multivariate ANOVA (PMANOVA) test on the Bray–Curtis dissimilarity matrix, implemented in the vegan v 2.5 (26) was used to test the effects (run, INO, C/F, and INO × C/F interaction with 9,999 permutations) on overall community composition.

## Results and Discussion

**Figure 1:**
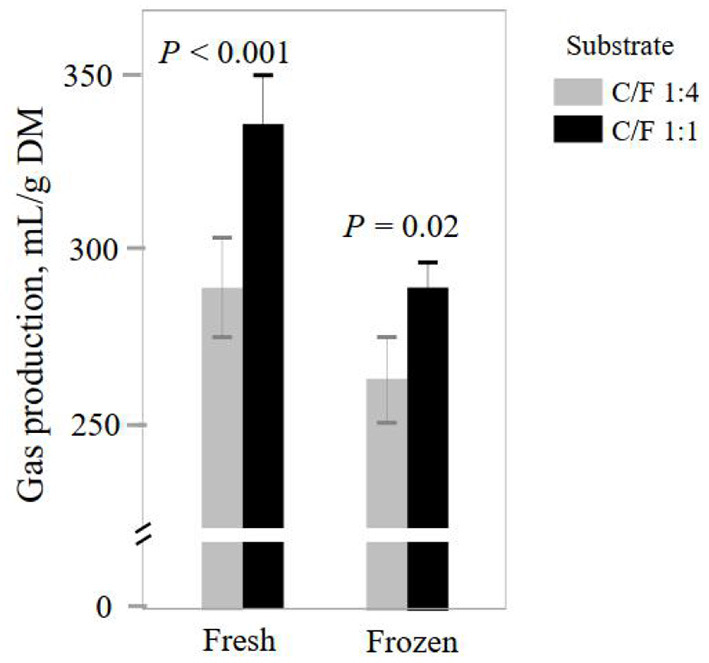
Effect of inocula (INO) and concentrate-to-forage ratio (C/F) on total gas production after 48 h *in vitro* rumen batch culture (*n* = 3).

*In vitro* degradation and gas production are important indicators for the efficiency of feed utilization by rumen microorganisms (1). Although both INO and C/F affected feed fermentation, and it did not alter ruminal pH (*P* ≥ 0.69, Table 1). Such unchanged pH can be caused by the well-buffered medium and help maintain the normal incubation process. Elevated C/F increased DM degradation and gas production (*P* ≤ 0.001; Table 1), which agrees with many previous studies (2, 12, 28). Such enhanced feed degradation is caused by the greater fermentation of starch than forage fiber (29). Frozen INO had lower DM degradation and gas production than fresh INO (*P* ≤ 0.001). Previous studies also report that frozen INO decreases DM degradation (10) or gas production (10, 30). Such inhibition of substrate degradation is reported to be caused by loss of microbial diversity or activity and can be avoided through a quick frozen process with a high surface-to-volume ratio (31). However, INO × C/F interaction was not observed for feed degradation expressed as DM degradation and gas production per g DM degraded, indicating independent effects of INO and C/F. We found an INO × C/F interaction effect for gas production expressed as ml/g DM (*P* = 0.04), and both fresh and frozen INO yielded greater gas production expressed as ml/g DM in higher C/F treatment (*P* ≤ 0.02, Figure 1). Although frozen INO caused a reduction in feed degradation, it can be employed to distinguish the effect of C/F on rumen feed degradation.

**Table 1:** Effect of inocula (INO) and concentrate-to-forage ratio (C/F) on rumen degradation and fermentation after 48 h *in vitro* rumen batch culture (*n* = 3).

**Items**	**INO**		**C/F**		**SEM**	***P-*** **value** ^**a**^		
	**Fresh**	**Frozen**	**1:4**	**1:1**		**INO**	**C/F**	**INO × C/F**
DM degradation, %	69.4	65.1	57.8	67.7	0.38	<0.001	<0.001	0.67
**Gas production**								
mL/g DM	328	294	276	312	2.6	<0.001	<0.001	0.04
mL/g of DM degraded	476	455	481	462	7.5	0.08	0.13	0.27
Total VFA (m*M*)	62.7	54.0	52.8	63.9	1.25	0.002	<0.001	0.43
pH	6.40	6.40	6.40	6.40	0.006	0.91	0.69	0.91
**The molar proportion of individual VFA, mol/100 mol**								
Acetate	67.0	67.3	68.3	66.0	0.49	0.64	0.01	0.78
Propionate	20.2	22.6	21.4	21.4	0.21	<0.001	0.98	0.85
Butyrate	7.90	6.34	6.37	7.87	0.363	0.01	0.01	0.77
*Iso*-butyrate	1.35	1.09	1.17	1.28	0.057	0.01	0.22	0.89
Valerate	1.29	1.08	1.04	1.32	0.054	0.02	0.006	0.77
*Iso*-valerate	2.15	1.46	1.61	2.01	0.104	0.001	0.02	0.98
**Acetate to propionate ratio**	3.30	2.97	3.19	3.08	0.024	<0.001	0.01	0.50

a *INO, inocula; C/F, concentrate-to-forage ratio; DM, dry matter*.

*In vitro* ruminal technique allows the estimation of net VFA production without the interference of the passage rate and absorption, which is a relevant method for nutritional evaluation of ruminant diets (32). In the current trial, both INO and C/F could affect production of VFA. Increasing C/F increased total VFA concentration with decreased acetate to propionate ratio (*P* ≤ 0.01, Table 1). Such results are in agreement with many published literature (2, 12, 28), as starch is more degradable and favors propionate production in comparison with fiber (33). Frozen INO decreased total VFA concentration and altered fermentation profile with decreased acetate-to-propionate ratio (*P* ≤ 0.001). Such reduction in net VFA production and change in rumen fermentation pathway can be caused by the less extent of fiber degradation in the treatment of frozen INO, which agrees with a previous study (11). However, the study indicates that differences in VFA profiles and DM degradation could be avoided when the INO is freezed quickly (31). Although the frozen INO altered rumen fermentation, the lack of INO × C/F interaction indicated independent effects of INO and C/F on total VFA concentration and VFA profile. These results suggest that the role of C/F on fermentation patterns can be successfully distinguished by inoculating frozen INO in *in vitro* fermentation.

**Figure 2:**
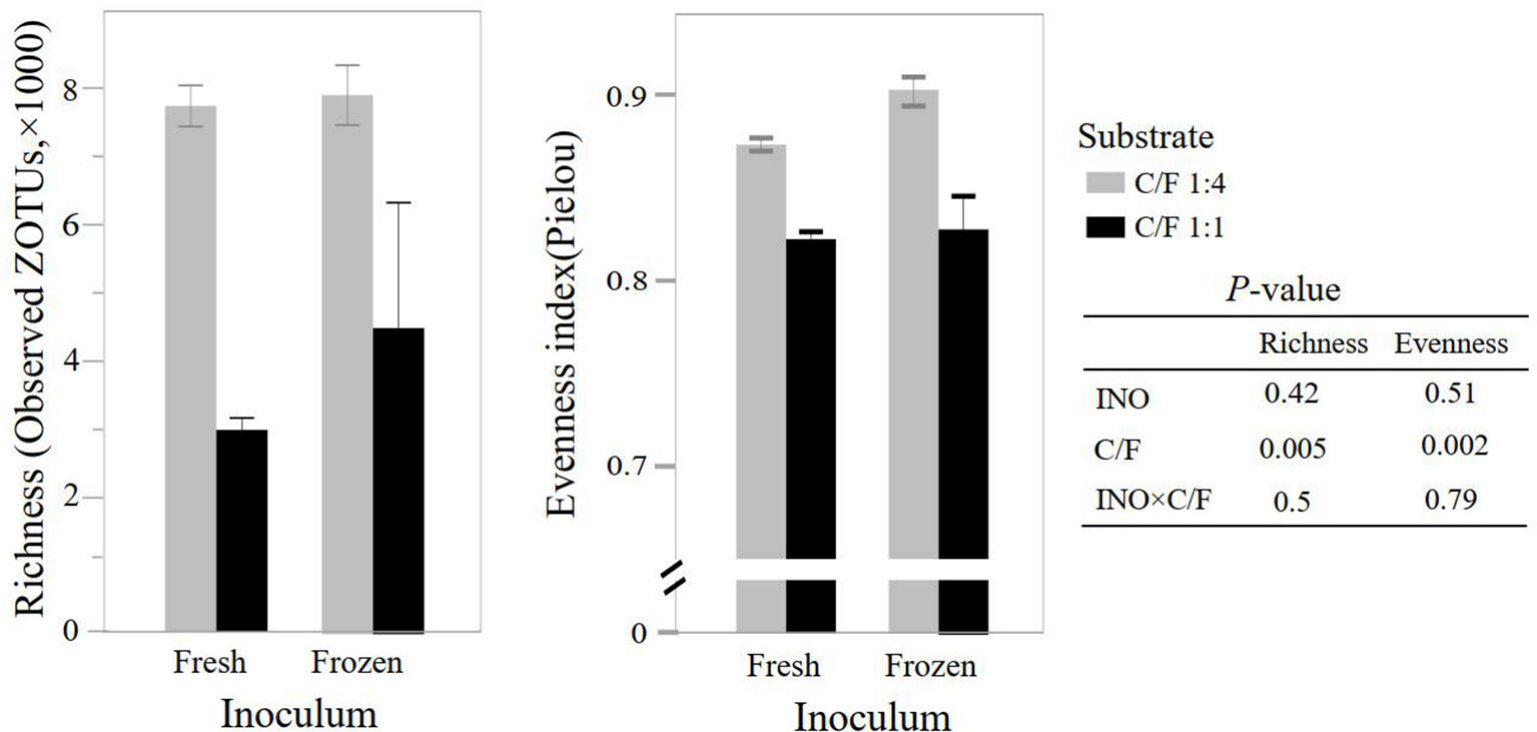
Effect of inocula (INO) and concentrate-to-forage ratio (C/F) on bacterial richness and evenness at zero-radius OTU (ZOTU) level after 48 h *in vitro* rumen batch culture (*n* = 3).

**Figure 3:**
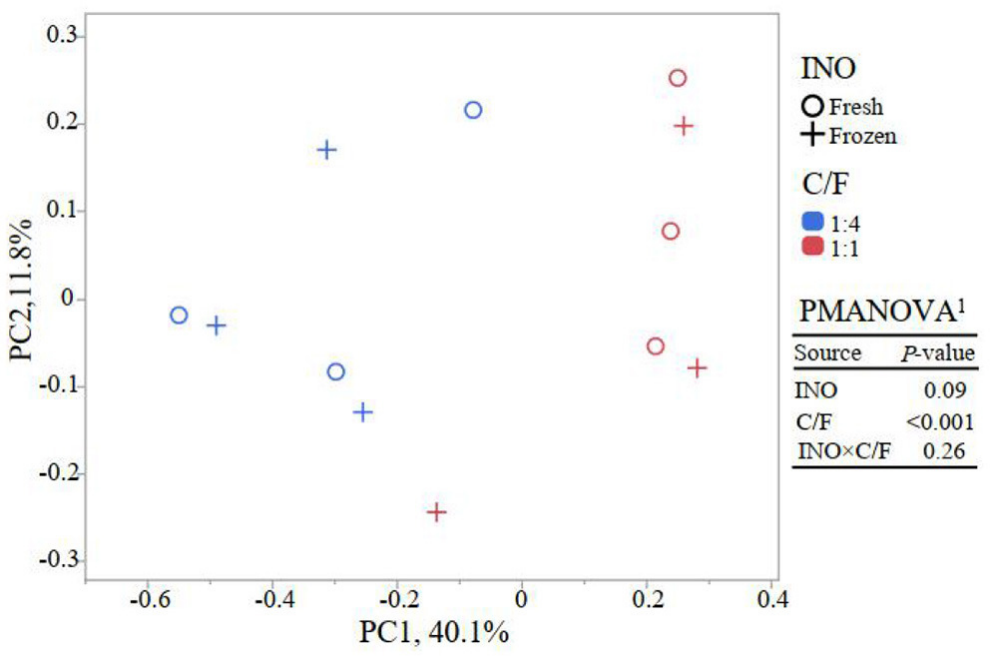
Principal coordinate analysis (PCoA) of bacterial community base on Bray–Curtis dissimilarity matrix at the OTU level (*n* = 3). ^1^PMANOVA, permutational multivariate ANOVA; INO, inocula; C/F, concentrate-to-forage ratio.

Although frozen INO reduced feed degradation and changed the fermentation pathway, it had little effect on the richness and evenness of the rumen bacterial community (*P* ≥ 0.42, Figure 2). This result indicated that even with lower activity, the major bacteria remained in the *in vitro* culture buffers. The INO had a tendency (*P* = 0.09) to affect the overall bacterial community estimated by the Bray–Curtis dissimilarity matrix. We speculated that the DNA of the inactive or even dead microorganisms remained in the fermentation flask, considering that our fermentation fluids and substrates were never renewed as they were *in vivo*. Furthermore, the INO × C/F interaction did not affect bacterial richness and evenness (*P* ≥ 0.50, Figure 2), and the overall bacterial community (*P* = 0.26, Figure 3). Regardless of the effect of INO, increasing C/F expectedly affected alpha diversities with a reduction in both richness and evenness (*P* ≤ 0.005, Figure 2), which is in agreement with many published literature (13, 34, 35). Moreover, the PcoA indicated that samples were clustered according to C/F other than INO (Figure 3). Both alpha and beta diversities indicated that the overall bacterial communities were weakly changed by INO, but were distinct in increasing C/F substrates.

Although frozen INO did not change overall microbial diversity, it still affected the abundance of some groups of bacteria. Frozen INO increased the abundance of acetate-producing genus *Ruminococcus* (*P* = 0.007, Table 2). Such change is out of our expectation since frozen INO did not alter the molar proportion of acetate. It has been reported that *Ruminococcus flavefaciens* can be isolated from frozen rumen liquid (36). Possibly, *Ruminococcus* spp. can recover faster from freeze-shock than others. Furthermore, frozen INO showed a tendency to decrease abundance of genus *Bacteroidales_*BS11 (*P* = 0.10) and *Christensenellaceae*_R-7 (*P* = 0.09). Although no strain from genus *Bacteroidales_*BS11 has been isolated, recent metagenomic techniques reveal that they have functions to degrade hemicellulose into acetate and butyrate (37). The abundance of genus *Bacteroidales_*BS11 is positively correlated with the molar proportion of butyrate (38). Genus *Christensenellaceae*_R-7 belongs to the family *Cristensenellaceae*, of which isolated strain *Christensenella minuta* sp. nov. can produce acetate and butyrate (39). We suspected that members in genus *Christensenellaceae*_R-7 might have functions to produce butyrate. The reduction in the abundances of genera *Bacteroidales_*BS11 and *Christensenellaceae*_R-7 may explain the decrease in the molar proportion of butyrate caused by frozen INO.

**Table 2:** Effect of inocula (INO) and concentrate-to-forage ratio (C/F) on abundance (%) of the taxonomy of bacterial community after 48 h *in vitro* rumen batch culture (*n* = 3).

**Taxonomy level^**a**^**	**INO**		**C/F**		**SEM**	***P*** **-value** ^**b**^		
	**Fresh**	**Frozen**	**1:4**	**1:1**		**INO**	**C/F**	**INO × C/F**
***Bacteroidota***	**37.6**	**31.9**	**21.8**	**47.6**	**2.25**	**0.36**	**0.005**	**0.45**
*Rikenellaceae_RC*	10.3	8.0	5.2	13.0	1.39	0.28	0.007	0.37
F082	5.81	4.82	2.88	7.75	1.115	0.55	0.02	0.20
*Prevotella*	4.94	5.56	2.61	7.89	1.114	0.70	0.01	0.27
*Bacteroidales_*BS11	3.84	1.51	1.13	4.22	0.863	0.10	0.04	0.13
*Muribaculaceae_*ge	2.15	1.96	1.21	2.89	0.383	0.73	0.02	0.35
*Bacteroidales_*UCG-001	1.34	1.78	1.08	2.04	0.307	0.34	0.06	0.30
***Firmicutes***	**22.5**	**19.3**	**13.3**	**28.5**	**2.46**	**0.38**	**0.004**	**0.31**
*Christensenellaceae_R-7*	2.68	1.89	1.29	3.28	0.289	0.09	0.02	0.23
*Lachnospiraceae_*unclassified	1.49	1.94	1.08	2.35	0.403	0.45	0.06	0.85
*Ruminococcus*	0.58	1.87	0.92	1.54	0.230	0.007	0.10	0.80
*Succiniclasticum*	1.34	0.77	0.72	1.39	0.238	0.14	0.09	0.40
*Oscillospirales_*ge	1.16	0.83	0.62	1.37	0.143	0.15	0.01	0.46
***Proteobacteria***	**13.9**	**18.2**	**24.9**	**7.2**	**2.89**	**0.33**	**0.005**	**0.41**
*Gammaproteobacteria_*unclassified	3.04	5.12	6.74	1.42	0.964	0.17	0.008	0.72
*Chromatiaceae_*unclassified	1.07	0.95	2.02	0.009	0.342	0.80	0.006	0.78
***Desulfobacterota***	**4.72**	**6.02**	**9.62**	**1.12**	**1.120**	**0.44**	**0.001**	**0.82**
*Desulfobacterales_*unclassified	0.66	1.02	1.58	0.10	0.186	0.21	0.003	0.54
***Acidobacteriota***	**2.38**	**3.26**	**4.73**	**0.90**	**0.719**	**0.41**	**0.009**	**0.64**

a *Top 5 phyla and their affiliated genera with an abundance higher than 1% were presented*.

b *INO, inocula; C/F, concentrate-to-forage ratio*.

Elevated C/F increases substrate degradation and gas production, but decreases acetate-to-propionate ratio and bacterial diversity. Such typical C/F effects are independent of INO, although INO affects the rumen degradation and fermentation. However, it should be noted that generalizations of results from studies with a small number of animals to large populations need to be cautious. In any event, under the conditions used in this experiment, we could suggest that liquid nitrogen frozen rumen fluids may potentially be used as *in vitro* microbial INO to validate the effects of changing the C/F in the diet on rumen fermentation and bacterial community.

## Data Availability Statement

The datasets presented in this study can be found in online repositories. The names of the repository/repositories and accession number(s) can be found below: https://www.ncbi.nlm.nih.gov/; bioproject/PRJNA748898.

## Ethics Statement

The animal study was reviewed and approved by Animal Care Committee, Institute of Subtropical Agriculture, The Chinese Academy of Sciences, Changsha, China.

## Author Contributions

MW, JZ, and SY were involved in the methodology and conceptualization. ZM prepared the original draft and data curation. MW reviewed and edited the manuscript. MW and ZT were involved in project administration. All authors have read and agreed to the published version of the manuscript.

## Funding

This work was supported by the Strategic Priority Research Program of the Chinese Academy of Sciences (XDA26040203), National Natural Science Foundation of China (Grant Nos. 31922080, 32002204, and 31730092), Hunan Province Science and Technology Plan (2020NK2066 and 2022NK2021), Innovation Promotion Association CAS (Grant No. Y202078), China Agriculture Research System of MOF and MARA, and Open Fund of CAS Key Laboratory of Agro-Ecological Processes in Subtropical Region (No. ISA2021203). The funders had no role in the design of the study; in the collection, analyses, or interpretation of data; in the writing of the manuscript, or in the decision to publish the results.

## Conflict of Interest

The authors declare that the research was conducted in the absence of any commercial or financial relationships that could be construed as a potential conflict of interest.

## Publisher's Note

All claims expressed in this article are solely those of the authors and do not necessarily represent those of their affiliated organizations, or those of the publisher, the editors and the reviewers. Any product that may be evaluated in this article, or claim that may be made by its manufacturer, is not guaranteed or endorsed by the publisher.
